# Multicenter clinical trial for the treatment of obstructive sleep apnea with a non-permanent orthodontic intraoral device in children

**DOI:** 10.1007/s00431-025-06254-x

**Published:** 2025-06-17

**Authors:** Clete A. Kushida, James Stevens, Michael Bennett, Tammarie Heit, Dennis Klemp, Dean Raio, Jesse Cozean, Colette Cozean

**Affiliations:** 1 Stanford University Division of Sleep Medicine, MC 5704, 430 Broadway Street, Pavilion C, 2nd Floor, Redwood City, CA 94063-5704 USA; 2 Stevens Health Alliance, 922 E. Edison Avenue, Sunnyside, WA 98944 USA; 3 Advanced Dental Care, 247 W 2230 N, Suite 101, Provo, UT 84604 USA; 4 Uptown Dental Centre, 10665 Jasper Avenue, Unit 106, Edmonton, AB T5J 3S9 Canada; 5 Klemp Family Dentistry, 1006 W Marine Dr, Astoria, OR 97103 USA; 6 Raio Dental, 1739 N Ocean Avenue, Medford, NY 11763 USA; 7 The EyeDeas Company, 21581 Midcrest Drive, Lake Forest, CA 92630 USA

**Keywords:** Pediatric obstructive sleep apnea, Oral appliance, Orthodontic device

## Abstract

**Abstract:**

The purpose of this study is to evaluate the safety and efficacy of a non-permanent orthodontic oral appliance intermittently worn in reducing the signs and symptoms of pediatric obstructive sleep apnea (OSA). A non-randomized interventional pre-post study was conducted in 2018–2021 with up to 24-month follow-up at five US and Canadian sites. Participants were 55 enrolled OSA-diagnosed children, fit with a customized device worn in the evening and while sleeping to provide slow maxillary expansion. Participants were assessed for treatment efficacy by three co-primary endpoints, the Pediatric Sleep Questionnaire (PSQ), apnea-hypopnea index (AHI), and intermolar width; and two secondary endpoints, the PSQ Sleep-Related Breathing Disorders (SRBD) subscale scores and airway volume by cone-beam computed tomography (CBCT) scans. Forty-seven participants were included in the analytic dataset following trial completion after 12–24 months. PSQ symptom scores decreased 31.0% with a posttreatment-pretreatment difference score mean±SE and 95% CI of −0.13 ± 0.019 [−0.17, −0.09]; AHI decreased 29.3% (−3.47±1.015 [−5.52, −1.42]). All participants showed an intermolar width increase, averaging 13.0% (4.03 ± 0.421 [3.18, 4.88]). PSQ SRBD subscale scores decreased 57.8% (−0.178 ± 0.031 [−0.24, −0.12]). Airway volume by CBCT scans increased 67.8% (4053.78 ± 885.433 mm^3^ [2265.62, 5841.95]). There were no safety concerns.

*Conclusion*: Participants showed objective and subjective OSA improvement, and all demonstrated maxillary expansion. Seventy-nine percent of participants showed AHI improvement, with 61.7% improving by 50% or more, and 17% resolved their OSA. Seventy-seven percent with moderate or severe OSA improved by 50%, while 93% with severe OSA achieved this milestone. This is the first study demonstrating that slow maxillary expansion by this device is safe and efficacious in treating children with OSA.

*Trial registration*: ClinicalTrials.gov ID NCT05661747 (https://clinicaltrials.gov/ct2/show/NCT05661747) Registered: December 14, 2022, retrospectively registered.

**Table:** 

**What Is Known:** *• Adenotonsillectomy is the primary treatment for pediatric obstructive sleep apnea (OSA) but is invasive and has proximal and long-term risks. Secondary treatments include rapid maxillary expansion that has numerous adverse effects due to its 24/7 use and treatment speed, and positive airway pressure that is limited by patient intolerance and nonadherence.* **What Is New:** *• This oral appliance is the first one FDA cleared for OSA treatment in children, and this study showed that slow maxillary expansion by this device was safe and efficacious for treating pediatric OSA. It is also the only pediatric non-permanent orthodontic device that is removable and intermittently worn to treat OSA.*

**Graphical Abstract:**

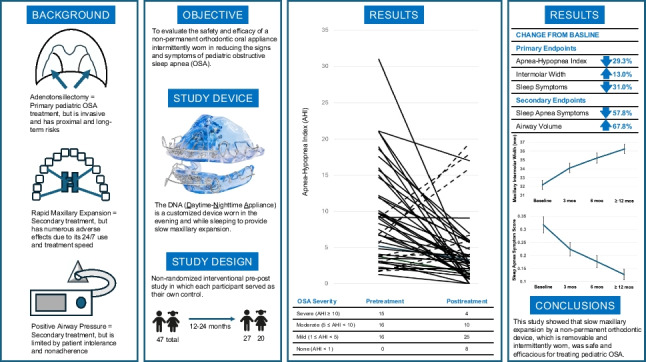

## Introduction

The American Academy of Pediatrics (AAP) in 2012 estimated that the prevalence of pediatric obstructive sleep apnea (OSA) is in the range of 1–5% and habitual snoring 1.5–27.6%, which are the most common disorders in the spectrum of sleep-disordered breathing (SDB) [1, 2]. Some conditions associated with or complicated by chronic SDB in children include neuropsychiatric and behavioral disorders, obesity, attention deficit/hyperactivity disorder, developmental delay, and cardiovascular disease [2]. The AAP’s recommendation is adenotonsillectomy as the first-line treatment for OSA in children [1]. However, for an apnea-hypopnea index (AHI; #abnormal breathing events/hour of sleep) ≥ 1, which meets criteria for pediatric OSA, 19–73% of patients had OSA that persisted after surgery [3]. An association of long-term risk for development of respiratory, allergic, and infectious diseases following childhood removal of adenoids and/or tonsils is also emerging [4]. For children in which surgery is contraindicated or unwanted, positive airway pressure (PAP) is recommended; however, approximately 30% are PAP nonadherent [5]. For those adherent to PAP, the average estimated duration children wear PAP devices each night is 5.5 h (about 55% of their sleep time) and PAP may be a challenge for children with conditions such as Down syndrome [3].

Oral appliances are recommended by the American Academy of Sleep Medicine (AASM) to treat adults with SDB, particularly OSA, when they refuse or are intolerant of PAP, as well as for snoring [6, 7]. The most common oral appliance is a mandibular advancement device (MAD) consisting of two linked trays that advance the mandible and tongue forward and result in anterior-posterior upper airway expansion when viewed cross-sectionally. A 2023 MAD systematic review and meta-analysis that included six studies with 180 children with OSA split into two groups (oral appliances and controls) showed that MAD significantly improved the AHI and enlarged the superior posterior airway space [8]. However, conventional oral appliances are a lifelong therapy that must be worn every night to effectively treat OSA.

The DNA (Daytime-Nighttime Appliance, Vivos Therapeutics, Inc., Littleton, CO) is an US Food and Drug Administration (FDA)-cleared product that uses slow maxillary expansion and is intended to reduce snoring and/or mild to moderate obstructive sleep apnea (OSA) in adult patients 18 years of age or older. The DNA and the mRNA (**m**andibular-**R**epositioning **N**ighttime **A**ppliance, an FDA-cleared combination device that adds an MAD to the DNA to treat mild-to-moderate OSA and snoring in adults) comprise the Complete Airway Repositioning and Expansion (CARE) approach to treat OSA by expanding the airway, improving sleep quality, and reducing OSA severity [9–13]. The DNA versus conventional oral appliances for OSA is unique in that the DNA is inserted and removed daily providing periodic expansion pressure, instead of an expansion appliance worn on a permanent basis. The DNA has recently been FDA cleared to treat moderate-to-severe OSA and snoring in children, which is the first time an oral appliance has been FDA cleared or approved to treat pediatric OSA.

This study evaluated the safety and efficacy of slow maxillary expansion with DNA in children with OSA.

## Materials and methods

### Study aims and design

The present study used a non-randomized interventional pre-post study design; each participant served as their own control with measurements taken before treatment (baseline) and then following treatment. The study was conducted with the purpose of evaluating the efficacy and safety of an orthodontic device that expands the pharyngeal airway. Specifically, the study aims were to assess if the device could reduce symptoms and signs of SDB and confirm treatment mechanism of action, as assessed by subjective and objective OSA severity and intermolar width, respectively. Additionally, airway volume was measured using cone-beam computed tomography (CBCT). The study is registered in ClinicalTrials.gov (https://clinicaltrials.gov/ct2/show/NCT05661747), the protocol was approved by the WCG Institutional Review Board, and the participants’ legally authorized representatives provided written informed consent to their study dentists before participation.

### Study device

**Fig. 1:**
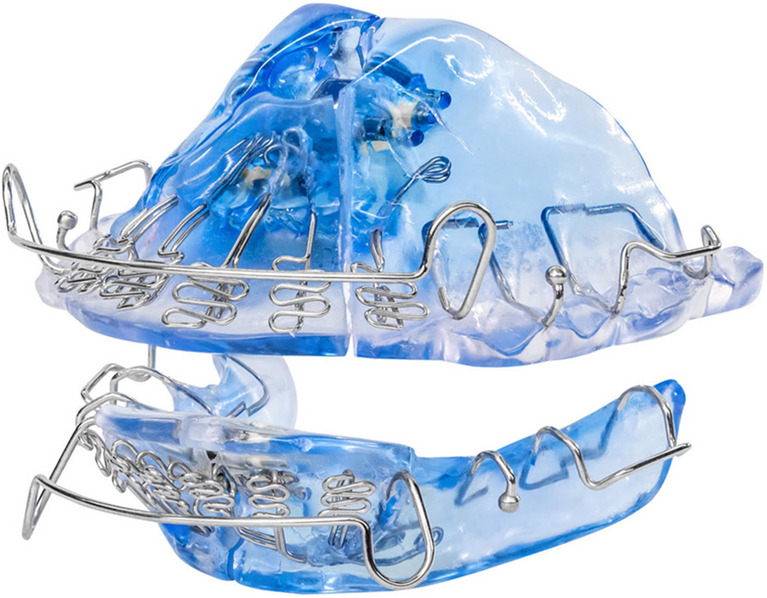
Daytime-nighttime appliance (DNA)

The DNA (Figure 1) is a customized oral appliance that is constructed from models of the patient’s teeth, using standard orthodontic acrylics and orthodontic wires for clasps and retention. The DNA may be adjusted antero-posteriorly, transversely, as well as permitting adjustments of the vertical dimension of occlusion. There is an optional extender on the device which further aids in opening the pharyngeal airway. The dentist prescribes the amount of acrylic to be utilized from a small amount of acrylic for a wire frame to complete coverage of the palate and the vertical dimension to meet the patient’s needs. At regular intervals (usually once a week, but at least once a month), the patient in most cases and/or dentist adjusts an expansion screw by 0.25 mm (or the dentist adjusts the loop) to expand the appliance as prescribed. The DNA is used in the evenings and at night by patients. Treatment usually lasts from 6 months to 24 months, but the dentist adjusts the treatment time as necessary to achieve the desired or stable results.

### Sites and participants

Five dental sites (four US sites and one Canadian site) agreed to participate in this clinical trial to confirm whether maxillary expansion with the DNA would improve SDB in children. Inclusion criteria consisted of age < 18 years, permanent teeth or mixed dentition at time of evaluation, diagnosis of sleep-disordered breathing (OSA AHI ≥ 1), in need of orthodontic treatment, and living in the USA or Canada. Exclusion criteria consisted of uncontrolled diabetes, any severe respiratory condition (e.g., chronic asthma, emphysema, and COPD), braces, and protocol nonadherence.

### Endpoints and measures

The three coprimary endpoints were as follows:

1. Total score on the Pediatric Sleep Questionnaire (PSQ). The PSQ is a validated questionnaire for determining sleep-related breathing disorders, snoring, sleepiness, and behavior in children and was administered to participants at pre- and posttreatment (final visit).
2. Sleep study-derived apnea-hypopnea index (AHI). Each participant received a home sleep apnea test (HSAT; Nox T3, Nox Medical, Reykjavík, Iceland or WatchPAT 300, ZOLL Medical Corporation, Caesarea, Israel) for pre- and posttreatment sleep studies at an AASM-accredited sleep center. The pretreatment sleep study was conducted within 1 year prior to the start of treatment. A sleep medicine physician not associated with the study and blinded to the pre- vs. posttreatment condition reviewed the HSAT data and assessed the participants’ degree of OSA, as they would for standard-of-care OSA management. WatchPAT 300 is cleared for ages ≥ 12 years (or > 65 pounds) and Nox T3 for ages ≥ 2 years. All children were tested with the same device at pre- and posttreatment (final visit).
3. Intermolar width. Using a digital vernier caliper, the minimum intermolar width was measured from the cementoenamel junction of the lingual necks of the right and left maxillary first molars to reduce effects of any possible tipping of the teeth; this width was measured 3 times for each participant with the average reported. Although mandibular measurements are also usually obtained, maxillary intermolar width was the relevant measure for this study and was obtained pre- and posttreatment (final visit) as well as intermediate timepoints from consistent anatomical landmarks. Manual intermolar width using digital vernier calipers are considered generally reliable; deviations in intermolar width measurements between hand-held digital vernier calipers and digital analyses have been found to be clinically insignificant [14].

The secondary endpoints were as follows: (1) sleep-related breathing disorders (SRBD) subscale of the PSQ, which was administered at the pre- and posttreatment (final visit) plus intermediate timepoints, and (2) airway volume from cone-beam computed tomography (CBCT). CBCT images were obtained from participants by the treating dentists or referred for capture at a qualified facility for pre- and posttreatment (final visit). All dentists were taught how to reproducibly conduct CBCT measurements following a standard protocol that included strict head positioning, with a field of view characterized as large (13 cm) that was the minimum field to visualize all the required anatomy determined by a scout acquisition. Airway volume was measured by CBCT at a defined region of the pharynx described in an earlier published article [13]. A copy of the CBCT images was sent to a certified radiologist for review and a qualified dental CBCT examiner for analysis and assessment; both CBCT reviewers were not associated with the study. The analysis was sent back to the treating dentist with detailed measurements, including airway size, shape, and capacity (with airway volume measured at the narrowest point); standard orthodontic measurements; and comparison of the participant’s measurements to population standards.

All of these endpoints were measured at pre- and at posttreatment (final visit). For the objective measures, they were conducted according to standard protocols and *without* the DNA in their mouths. Sleep studies and CBCT images were reviewed by outside experts, minimizing bias and enhancing reliability.

Additional measures included the following: (1) device use, in which an estimate of average hours per 24-hour period of device usage were obtained from each participant at each posttreatment visit, and (2) safety parameters, which were evaluated at each visit, and included pain, tissue swelling, molar tip buccally, mandibular rotation or open bite, gingival recession, temporomandibular joint disorder, root resorption, change in bone density, loose teeth, or other concerns.

### Device fabrication, fitting, use, and follow-up visits

The orthodontic assessment by the treating dentist was conducted according to orthodontic diagnosis by the American Dental Association (ADA). The dentist obtained photographs (both intraoral and portrait), diagnostic casts of upper and lower arches, and dental radiographs (e.g., a full mouth series as needed). The dentist sent a device prescription and used these data and analyses to develop a design individualized for the participant upon which the DNA would be fabricated by a Certified Dental Laboratory (CDL). The dentist fitted the participant with the DNA and provided instructions for its use and maintenance. The instructions specified that the DNA is designed to be worn for 16 h/day (minimum of 10 h/day) for optimal results, with the best time of use when sleeping and relaxed times in the late afternoon and evening at home. From the DNA fitting date, a follow-up visit for additional adjustment within the initial 24–72 h could be scheduled with the dentist, with a subsequent 2-week visit to check the device fit, and then every 3–4 weeks thereafter (as indicated by standard-of-care orthodontic treatment). Participants completed the PSQ SRBD subscale at visits 2 weeks; 3, 6, 12 months; and additionally at 18 and 24 months (depending on the duration required for completion), with device adjustments made based on symptoms and comments made by participants and/or parents/guardians. The dentist concluded the study at any point after 6 months and up to 24 months when the participant achieved the desired orthodontic results, due to complications, or for any reason (e.g., participant request, dentist decision). Upon study completion, the dentist obtained the final diagnostic data, including a posttreatment sleep study and analysis by a sleep medicine physician, CBCT and analysis by dental examiners, intraoral radiographs (as needed), diagnostic photographs (intraoral and portrait), diagnostic casts, and case report forms. All study tests and procedures are considered standard-of-care orthodontic treatment, except for the posttreatment sleep study and posttreatment CBCT.

### Treatment completion criteria

Participant treatment was complete when the following criteria were met:

1. Space for all mandibular incisors to line up straight with either orthodontic brackets or clear aligners
2. Sufficient intercanine width for maxillary incisors to line up straight
3. Subjective improvement in sleep quality and daytime fatigue as assessed by the PSQ
4. Objective improvement in airway volume and cross section and/or AHI from HSAT

### Data analysis and monitoring

#### Sample size estimate

With a power of 80%, a confidence level of 95%, alpha (possibility of Type I error) of 0.05, and an assumed improvement of 50%, it was estimated that approximately 40 participants would provide statistically significant results. At least 50 participants were needed to be enrolled in the study to allow up to 20% (*N* = 10) dropouts.

#### Data analysis

This analysis was conducted by pairing pretreatment measurements with posttreatment measurements at 3 months, 6 months, and final testing, which could be at 6, 12, 18, or 24 months. Paired *t*-tests were used to analyze pre- and posttreatment data for all coprimary and secondary endpoints. A linear mixed-effects model for repeated measures (MMRM) was used to analyze data for endpoints where intermediate timepoints were collected (intermolar width and SRBD subscale of the PSQ). Two separate models were constructed, one with intermolar width as dependent variable, and the other with SRBD subscale of the PSQ as dependent variable, each with fixed effects of time, age, gender, height, and weight. Missing data were imputed using expectation-maximization (EM) algorithms, with the exception of missing CBCT data from one site that did not conduct these scans on their five participants. Pearson correlations were obtained for the posttreatment-pretreatment difference scores for all endpoints and (1) participant age at pretreatment visit and (2) participant estimates of number of hours of use per 24-h period (averaged across visits). SPSS (IBM SPSS Statistics, Version 29) was used for all statistical analyses.

#### Data monitoring

Each dental site securely uploaded the data to an independent third party who monitored the study and conducted the data analyses. The sponsor was blinded to the results and the study dentists and physicians were blinded from each other, although they did collectively attend training and protocol review.

## Results

### Participant disposition and demographics

This clinical trial was conducted from October 11, 2018, to December 10, 2021. Fifty-five patients were enrolled to participate in this trial at five sites. Seven participants dropped out: 2 each at 3 sites and 1 at a fourth site. Their reasons for dropping out were to pursue other treatments (braces, 1 participant; adenotonsillectomy, 2 participants), family relocation (2 participants), and protocol nonadherence (2 participants). Forty-eight participants completed the trial. During the trial, one participant was identified who did not meet the inclusion criteria due to an AHI of 0.9. Although the participant continued in the study, their data were excluded from all analyses. One site did not conduct CBCT scans during the trial, so airway volume measures were not obtained on the five participants at this site (see “Data analysis” section). The pretreatment demographics of the 47 participants included in the analytic dataset were well balanced between male and female participants (Table 1), though most participants were male.

**Table 1:** Pretreatment demographics (mean ± SD [range])

	Total	Male (*N* = 27)	Female (*N* = 20)
Age (years)	10.5 ± 2.57 [4.5–14.8]	10.6 ± 2.52 [4.5–14.8]	10.4 ± 2.70 [5.8–14.6]
Height (in)	55.2 ± 6.87 [39.0–68.0]	55.6 ± 5.69 [47.0–68.0]	54.6 ± 8.32 [39.0–67.0]
Weight (lbs)	84.5 ± 33.07 [38.8–180.0]	84.2 ± 30.00 [39.1–140.0]	85.0 ± 37.62 [38.8–180.0]

Since most children have all permanent teeth between 10 and 12 years of age, the study likely had 15 children with mixed dentition (6–9 years), 21 in the midrange of 10–12 years, and 11 with permanent dentition. All children met the age criteria for the study, except one child who was included in the WatchPAT 300 cohort based on body weight. The child was age 10.5 years but weighed 77 pounds, which exceeded the WatchPAT 300’s weight indication of ≥ 65 pounds in children.

### Efficacy

#### Coprimary endpoints

This analysis was carried out by pairing pretreatment measurements with posttreatment measurements, with PSQ scores, AHI measurements, and intermolar width (Table 2).

**Table 2:** Coprimary and secondary outcome measures data at pre- and posttreatment final testing

Outcome measures	Pretreatment (Mean±SD)	Posttreatment (Mean±SD)	Difference (Mean±SE)	95% CI	% Change	Paired *t*-test results
PSQ score	0.28±0.135	0.14±0.104	−0.13±0.019	−0.17, −0.09	31.0%	*t*(46) = 7.774 *p* < 0.001
Apnea-Hypopnea Index (AHI)	9.13±6.655	4.31±4.614	−3.47±1.015	−5.52, −1.42	29.3%	*t*(46) = 4.234 *p* < 0.001
Intermolar width (mm)*	32.18±3.457	36.23±3.649	4.03±0.421	3.18, 4.88	13.0%	*t*(46) = −10.674 *p* < 0.001
PSQ SRBD Subscale	0.32±0.214	0.12±0.139	−0.178±0.031	−0.24, −0.12	57.8%	*t*(46) = 6.480 *p* < 0.001
CBCT scan-derived airway volume (mm^3^)	9698.79±6647.455	13,752.57±5837.255	4053.78±885.433 mm^3^	2265.62, 5841.95	67.8%	*t*(46) = −4.465 *p* < 0.001

*One hundred percent (100%) of participants increased their intermolar width

PSQ score showed a significant 31.0% decrease pre- to posttreatment, indicating improvement in overall sleep disorder symptom severity.

**Fig. 2:**
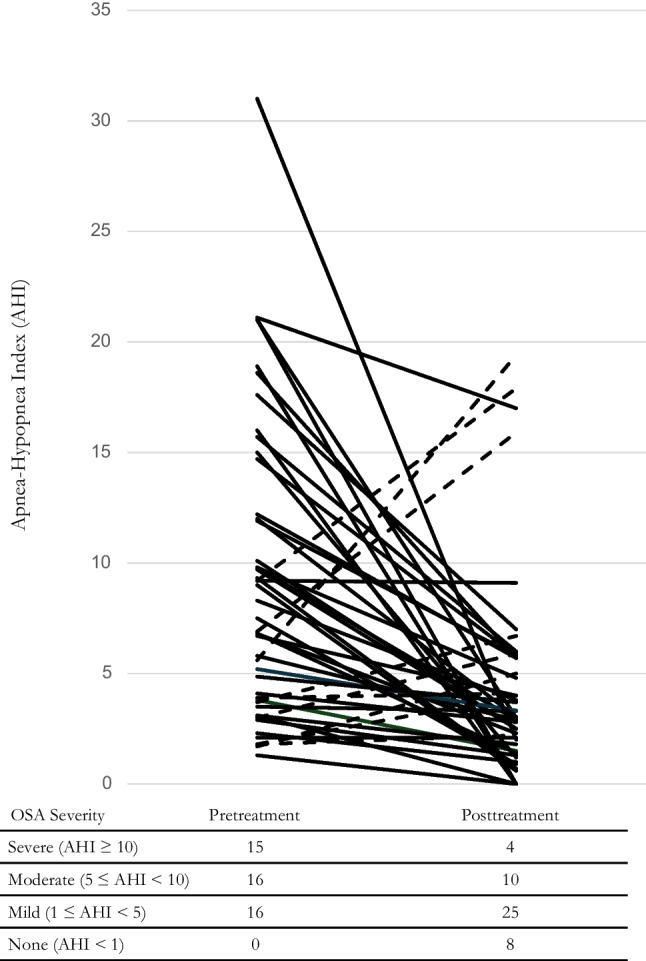
Apnea-Hypopnea Index (AHI) for each participant at pre- and posttreatment

Of the 47 participants, 37 (79%) had improved OSA severity by AHI, 9 (19%) worsened, and 1 (2%) showed no change (Figure 2); further, 61.7% had their AHI improved by 50% or more, 60.0% improved by one OSA severity classification, and 17.0% had their OSA resolved (AHI < 1). Of the 31 participants with moderate or severe OSA, 83.8% improved with a mean±SD percent change improvement of 67.0±37.50%. Of the 15 participants with severe OSA, 6 (40%) improved to moderate OSA, 5 (33%) improved to mild OSA, 3 (20%) had their OSA resolved, and 1 (7%) showed no change, resulting in 93% showing AHI improvement by 50% or more.

**Fig. 3:**
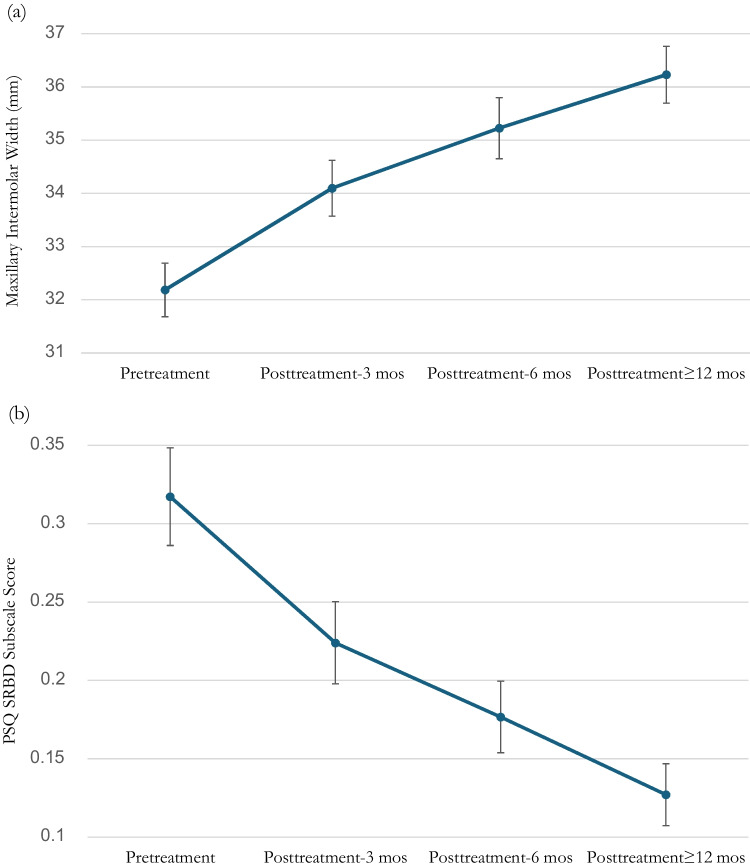
**a** Intermolar width (mean±SE) and (**b**) PSQ SRBD subscale score (mean±SE) over time

All participants showed an increase in intermolar width by an average of 13.0%. Intermolar width continued to almost linearly increase throughout the treatment (Figure 3a). Two participants met completion criteria in 6 months so their data were included in Figure 3a in the 6-month category. In the linear MMRM analysis, there were significant effects of time (*F*_3,180_ = 12.136, *p* < 0.001), gender (*F*_1,180_ = 25.520, *p* < 0.001), height (*F*_1,180_ = 5.940, *p* = 0.016), and weight (*F*_1,180_ = 5.657, *p* = 0.018), but not of age (*F*_1,180_ = 0.455, *p* = 0.501).

#### Secondary endpoints

The PSQ SRBD subscale scores and CBCT scan-derived airway volume showed significant improvement at posttreatment compared to pretreatment (Table 2). Similar to maxillary intermolar width (Figure 3a), there was an almost linear improvement in the PSQ SRBD subscale scores (Figure 3b). Both the PSQ SRBD subscale scores and CBCT-derived airway volume showed at least 50% improvement compared to pretreatment with 57.8% and 67.8% changes, respectively. In the linear MMRM analysis, there were significant effects of time (*F*_3,180_ = 10.499, *p* < 0.001) and age (*F*_1,180_ = 5.507, *p* = 0.02), but not of gender (*F*_1,180_ = 2.002, *p* = 0.159), height (*F*_1,180_ = 0.018, *p* = 0.892), and weight (*F*_1,180_ = 3.057, *p* = 0.082).

### Correlations

Significant correlations were found for the following: (1) participant age at pretreatment visit and the posttreatment-pretreatment difference scores for PSQ scores, *r* = −0.293, p = 0.046; and (2) participant estimates of number of hours of use per 24-h period averaged across visits and the posttreatment-pretreatment difference scores for CBCT-derived airway volume, *r* = 0.450, *p* = 0.003.

### Adjunctive modalities

No adjunctive modalities were utilized during treatment. Some of Vivos certified dentists believe in the use of myofunctional therapy for all pediatric patients. However, for this study, they did not use myofunctional therapy during the treatment period. It could have been used before or after treatment to train the tongue in proper positioning.

### Safety

There were no reports of safety issues, including pain, bone resorption, gum regression, or temporomandibular joint (TMJ) symptoms.

## Discussion

The DNA showed statistically significant (*p* < 0.01) efficacy for the three prespecified coprimary endpoints: subjective sleep improvement on the PSQ, objective OSA severity assessed by sleep study, and intermolar width. Statistically significant improvement was also shown for the secondary endpoints of the PSQ SRBD subscale scores and airway volume from CBCT. Intermolar width and PSQ SRBD subscale scores showed an almost linear improvement throughout treatment, and a moderate correlation was found for participant estimates of device use and objective increase in CBCT-derived airway volume. This is the first clinical study that demonstrates the efficacy and safety of a non-permanent orthodontic device in children for the treatment of OSA. There were no adverse effects reported for any of the participants.

Adenotonsillectomy is typically recommended for those children with OSA and enlargement of tonsils and adenoids, with PAP as second-line treatment. For those prepubertal children with a narrow palate and OSA, rapid maxillary expansion (RME) is an orthodontic treatment option, since the mid-palatal suture can be expanded more easily in children than adults. RME involves a downward and forward movement of the maxillary complex resulting in improved tongue posture (i.e., moving the tongue upward and closer to the palate) [15] as well as increased nasal airway caliber and airflow [16], with subsequent persistent improvement in OSA severity in multiple studies 0.3, 1, 3, and 12 years following RME [17–20]. In contrast to slow maxillary expansion of 0.25 mm of expansion/week, RME typically involves up to 0.5 mm of expansion/day, with a published average increase of 4.4 mm [21] that is in line with the results of our study. RME and slow maxillary expansion in children have not been evaluated in head-to-head comparative effectiveness studies, particularly since it is uncertain whether there are long-term benefits of expansion [22] and if they persist into adulthood [23]. However, it would be expected they would demonstrate similar degrees of OSA improvement and long-term efficacy given their similar mechanisms of action. The DNA in the current study used slow maxillary expansion and is not worn 24/7; due to its use only in the evening and nighttime, many of the numerous RME adverse effects [24, 25] are absent, including experiencing much less pain, but with preservation of key effects such as resumption of nasal vs. obligate mouth breathing.

The study had some limitations. The sleep studies were not in-laboratory sleep studies (polysomnography), and presently, polysomnography is recommended for childhood OSA diagnosis by the AASM in their 2017 position paper [26]. In the 8 years following these recommendations, numerous studies examined prospective and retrospective pediatric cohorts with HSATs, the majority of which support the feasibility and validity of using these devices to diagnose OSA in children. The recommendations are also in contrast to a 2023 guideline by the British Thoracic Society [27] (which allow HSATs in children without comorbidities) and a 2023 expert consensus statement by the American Academy of Otolaryngology-Head and Neck Surgery [28] (which allows HSATs when a PSG is not readily available or tolerated). Thus, given these points, the FDA approval of these HSAT devices (WatchPAT 300 and NoxT3) for the age range of the children in the present study, and that the same HSAT device was used for each participant’s pre- and posttreatment sleep study, we believe that the use of HSATs is valid in this trial. Another limitation is the choice of a non-randomized interventional pre-post study design, instead of a randomized controlled trial (either sham or treatment withdrawal). However, we believe that there are ethical and clinical equipoise considerations [29] in this latter type of study design, especially in a pediatric population. An additional limitation is that goniometry was not used to assess molar tipping that is a side effect of both slow and rapid maxillary expansion. RME can induce a hyaline zone around the maxillary teeth that helps to reduce molar tipping; hyaline zone induction has not been reported with slow maxillary expansion, although molar tipping has been reported to be less pronounced with slow expansion [30]. Lastly, although final testing following the DNA fitting date was up to 24 months, long-term assessment was not conducted past this timepoint.

The DNA achieved efficacy for the three coprimary endpoints without the device in the oral cavities of the children participating in the study. The non-permanent nature of the device has distinct advantages over conventional oral appliances, and it was well-tolerated without significant adverse effects.

## Conclusion

Slow maxillary expansion by this device was safe and efficacious for treating children with OSA and symptoms of sleep-disordered breathing. The DNA was inserted intermittently in the evenings and at night during sleep. Like other orthodontic treatments (except that outcome efficacy is assessed without the device in the mouth), it is expected that the treatment will have a permanent effect past the maximum 24-month study duration. Compared to CPAP, adenotonsillectomy, and RME, which have limitations such as adherence, complications, and 24/7 use, respectively, DNA offers a new option for pediatric OSA treatment.

## Data Availability

The data that support the findings of this study are not openly available due to reasons of sensitivity and are available from the corresponding author upon reasonable request. Data are located in controlled access data storage at the Stanford University School of Medicine.
